# Social media use and subjective well-being among older adults: chain mediation effects of future time perspective and social support

**DOI:** 10.3389/fpsyg.2026.1831189

**Published:** 2026-05-28

**Authors:** Zhuliu Gong, Yi Guo

**Affiliations:** School of Journalism and Communication, Chongqing University, Chongqing, China

**Keywords:** future time perspective (FTP), older adults, social media usage, social support (SS), socioemotional selectivity theory (SST), subjective well-being (SWB)

## Abstract

**Purpose:**

Based on socioemotional selectivity theory, this study examined the association between social media use and subjective well-being among older adults, focusing on the chain mediating roles of future time perspective and perceived social support.

**Methods:**

A cross-sectional survey using stratified sampling was conducted among 4,872 older adults (aged 60+). Structural equation modeling and bias-corrected Bootstrap were used for mediation analysis.

**Results:**

Social media use was positively associated with subjective well-being (*β* = 0.115, *p* < 0.001). Two independent mediating pathways were identified: future time perspective (effect = 0.055, 95% CI [0.043, 0.067]) and perceived social support (effect = 0.036, 95% CI [0.027, 0.046]). A chain mediation pathway was also significant (effect = 0.010, 95% CI [0.006, 0.014]), indicating that social media use was associated with well-being through its link with future time cognition, which in turn was associated with perceived social support.

**Conclusion:**

Digital participation may function as a psychological compensation mechanism associated with temporal cognition and social support. This study reveals pathways linking social media use to older adults’ well-being, offering theoretical and practical insights for active aging.

## 1 Introduction

Global society is experiencing the deep intersection of population aging and digital transformation. China’s aging process is particularly rapid, with the population aged 60 and above increasing from 178 million in 2010 to 310 million in 2024, representing a rise from 13.26 to 22% of the total population, marking entry into the moderate aging stage (Ministry of Civil Affairs and China National Committee on Aging, 2023). Simultaneously, internet participation among older adults is growing rapidly, with the number of netizens aged 60 and above in China surpassing 156 million, representing 14.1% of the total netizen population [China Internet Network Information Center (CNNIC), 2025]. In this context, digital technology has increasingly integrated into the daily lives of older adults, and how to promote their physical and mental health through digital participation has become an urgent issue for academia to address.

Within the active aging theoretical framework, subjective well-being is a core psychological indicator for measuring quality of life among older adults (Chan, 2018). Existing research has preliminarily confirmed a positive association between social media use and subjective well-being among older adults (Cotten et al., 2022; Viklund and Forsman, 2022). However, current research primarily examines the direct relationship between media use and subjective well-being among older adults, with relatively limited attention to potential mediating mechanisms (Nakagomi et al., 2022; Rosenberg and Taipale, 2022). Some studies have attempted to address this research gap by examining the mediating roles of social relationships, social inclusion, and social support (Yu et al., 2021; Nebenzahl-Elitzur et al., 2026; Hu and Tan, 2025), but exploration of psychological mechanisms remains insufficient (Zhang et al., 2025). Understanding psychological mechanisms is crucial because subjective well-being is essentially a product of positive psychological experiences, and its generation process is significantly influenced by specific psychological processes.

Among possible psychological mechanisms, two variables deserve attention. First is Future Time Perspective (FTP), which refers to individuals’ subjective perception of remaining lifetime and has been proven to be significantly related to subjective well-being among older adults (Li and Siu, 2021). Second is Social Support (SS), which, as one of the most important psychosocial resources for older adults, has been widely confirmed for its contribution to well-being (Su et al., 2022). Notably, socioemotional selectivity theory (SST) suggests that there may be a progressive association between these two variables: future time perspective influences social behavioral patterns by regulating social goals (Lang and Carstensen, 2002), and changes in social behavioral patterns are directly related to the level of social support acquisition. This implies that future time perspective and social support may not be independent parallel mediators in the process by which social media use is associated with older adults’ well-being, but rather constitute a chain mediation pathway from temporal cognition to social resources.

However, it seems no research has yet integrated social media use, future time perspective, and social support into a comprehensive theoretical model to systematically examine their joint mechanisms. Based on this, the present study uses SST as the core theoretical framework, integrating social support theory to construct a theoretical model with social media use as the independent variable, future time perspective and social support as chain mediators, and subjective well-being as the dependent variable, aiming to explore the pathways within the model.

## 2 Literature review and hypotheses

With the process of mediatization, scholars have gradually recognized the influence of media on shaping older adults’ subjective perceptions, including attitudes toward new technologies, willingness to accept new technologies, and overall subjective well-being (Yang et al., 2024; Hofer, 2016; Cotten et al., 2022; Viklund and Forsman, 2022). However, the academic field has not yet reached consensus on the relationship between social media use and residents’ subjective well-being, mainly presenting two competing perspectives: “positive enhancement” and “negative displacement.”

Researchers holding negative views point out that older adults face challenges such as resistance to internet technology and potential health risks associated with use, which may affect their subjective well-being across multiple dimensions including pleasure, personal growth, sense of control, autonomy, and social connection (Bianchi, 2021). For example, Kraut and Burke (2015) argued that ineffective communication with strangers and other weak ties during internet use often displaces deep communication time with family and close relatives (strong ties), and this misallocation of social resources actually exacerbates individuals’ loneliness and may even trigger depressive symptoms.

However, optimistic researchers believe that lifestyle changes triggered by internet technology contribute significantly to the psychological well-being of older adults. The internet breaks down spatial and temporal barriers, greatly expanding older adults’ social networks. On one hand, older adults maintain close contact with children, relatives, and friends through the internet, which helps them overcome temporal constraints and increase the frequency of face-to-face interactions, and this is positively associated with well-being (Yang et al., 2021; Braun, 2013). On the other hand, social media breaks down physical barriers to interpersonal communication, providing efficient pathways for social interaction. It is particularly effective in consolidating initial relationships and maintaining distant friendships, promoting the establishment and maintenance of interpersonal relationships with unprecedented convenience and creating a sense of belonging for users (Vitak and Ellison, 2012). Consequently, it is significantly associated with higher subjective well-being among older adults (Song et al., 2021). Furthermore, online activities, including social media use, can be viewed as forms of online social activity (Schehl et al., 2019), such as online learning, entertainment, and information searching. Activity theory suggests that older adults’ engagement in adequate social activities can maintain their connection with society, thereby contributing to subjective well-being (Havighurst, 1961; Jin et al., 2023).

Increasing evidence shows a significant positive correlation between social media use and improved well-being among older adults (Cotten et al., 2022; Harriger et al., 2023). A recent study found that the use of short video digital media is associated with higher subjective well-being among older adults mainly through multiple pathways of emotional satisfaction, flow experience, and habit formation (Li X. et al., 2025). Another more refined study found that the intensity of forwarding behavior in social media use has a significant positive predictive association with subjective well-being among older adults. The forwarding function, as a communication bridge for information dissemination, can not only simplify daily affairs processing and alleviate social avoidance tendencies, but also be linked to subjective well-being by promoting social participation (Wang and Xu, 2024). Based on the above, this study proposes the following research hypothesis:

*H*1: Social media use is positively associated with subjective well-being among older adults.

Previous research indicates that despite declining physical and cognitive functions with aging, older adults’ well-being levels are often higher than those of younger people. Socioemotional selectivity theory explains this well-being paradox: the limited perception of future time perspective becomes an antecedent variable for maintaining or even enhancing well-being through adaptive emotional regulation mechanisms. Socioemotional selectivity theory was proposed by Carstensen (1993) and has been developed and refined over more than 20 years, essentially being a motivational lifespan theory (Ao et al., 2011). This theory is built on three basic assumptions: first, social interaction is central to human survival, driving the continuous evolution of social interests and social attachments; second, human behavior is guided by expected goals; third, people hold multiple or even opposing goals, and goal selection precedes action and is influenced by time perception (Carstensen et al., 1999). Accordingly, socioemotional selectivity theory establishes future time perspective as the core mechanism explaining social goal transformation.

Numerous studies show that future time perspective affects subjective well-being among older adults (Ramsey and Gentzler, 2014; Li and Siu, 2021; Zhang et al., 2019; Lu, 2022). Future time perspective originally referred to individuals’ subjective perception of remaining lifetime, later expanded to the perception of time limits for any event’s conclusion; the prominence of endpoint awareness triggers individuals’ perception of time’s limitedness, and this limited or unlimited perceptual orientation predicts the priority order of social goals (Xing et al., 2013). Time perspective is defined as an unconscious psychological process through which individuals attribute continuous personal and social experiences to different temporal frameworks, thereby giving events order, coherence, and meaning; future time perspective is a behavior-oriented pattern facing the future (Zimbardo and Boyd, 1999; Stolarski et al., 2015; Rudolph et al., 2018). According to socioemotional selectivity theory, when future time is perceived as infinitely vast, knowledge acquisition goals dominate, driving information collection, novel experiences, and social network expansion behaviors; when future time is perceived as limited and contracting, emotion regulation goals become priority pursuits, with individuals focusing more on emotional states, emotional meaning, and immediate satisfaction (Carstensen, 1993; Charles and Carstensen, 2010). According to empirical research, future time perspective consists of two relatively independent factors, respectively focusing on future opportunities (FTP-opportunities) and focusing on time constraints (FTP-limitations) (Cate and John, 2007). These two factors can represent unique meanings of future perception (Cate and John, 2007; Kozik et al., 2015). As age increases, future time perspective gradually reduces, and individuals’ social goals systematically shift from knowledge to emotion. This motivational shift profoundly shapes social behavioral patterns among older adults: when time limitation becomes prominent, individuals, in pursuit of emotional meaning, tend to prioritize interaction with familiar social partners and actively reduce social networks to eliminate secondary relationships (Ao et al., 2011; Fung et al., 2008).

Mass media may already be shaping people’s perception of time, which in turn is associated with older adults’ subjective well-being. Lukyanova et al. (2020) studied factors influencing temporal behavior in online media, revealing the reshaping effect of internet environments on individual time perception. Görland (2019) research showed that mobile media use changes individuals’ time experience during daily interstitial periods. Wittkower (2010) further pointed out that social media enables us to view and share past memories at any time point, while also pulling us toward an eternal present, a phenomenon he termed “time flattening.” Some research indicates that social networks may be associated with older adults’ subjective well-being through the FTP-opportunities dimension of future time perspective (Zhang et al., 2019). Based on this, we propose that social media may expand older adults’ FTP-opportunities dimension through continuous cognitive stimulation and novel experiences, which in turn may be associated with their subjective well-being. These studies collectively indicate that digital media constitutes an important situational force reshaping individual temporal cognition. However, the specific pathways linking social media use, older adults’ future time perspective, and subjective well-being have not been systematically examined. Based on the above, this study proposes the following research hypothesis:

*H*2: Future time perspective mediates the association between social media use and subjective well-being among older adults.

Among factors affecting subjective well-being in older adults, social support occupies an irreplaceable position (Li Z. et al., 2025; Su et al., 2022; Yuan et al., 2025). From a conceptual perspective, social support refers to assistance individuals receive from others in social interactions. Based on functional differences, it can be divided into different types such as informational support, emotional support, instrumental support, social network support, and self-esteem support (Cutrona and Suhr, 1992; Xu and Burleson, 2001), with informational support, emotional support, and instrumental support being most common (Ke et al., 2010). Furthermore, the core meaning of social support lies in making individuals confident that they are cared for, loved, respected, and belong to a social network with reciprocal obligation relationships (Cobb, 1976).

In the Chinese cultural context, family support is particularly significant for subjective well-being among older adults. Due to the gradual reduction of original social connections caused by retirement, support from family members becomes the core pillar of older adults’ social support systems (Song et al., 2021). Compared to Western populations, family support has a more significant alleviating effect on depressive emotions among Asian older adults (Mohd et al., 2019). Some scholars even believe that intergenerational support is more important for Chinese older adults’ subjective well-being than social support from outside the family (Peng et al., 2015).

As digital technology deeply embeds in older adults’ daily lives, the mechanism through which social media use affects their subjective well-being has attracted widespread attention. Existing research suggests that internet use does not directly affect subjective well-being; social support is the potential linking mechanism between the two (Gu et al., 2025; Nam, 2021). From a social network theory perspective, internet use increases opportunities and pathways for individual self-expression, helping to expand social circles and obtain social support, which is in turn associated with higher subjective well-being (Oshio et al., 2020). Specifically, social media compensates for social support deficits caused by physical limitations through information retrieval functions, improving self-efficacy and thereby being associated with higher subjective well-being (Wei and Chang, 2026). More detailed mechanism analysis shows that perceived social support plays a key mediating role in this process. Research finds that the forwarding intensity of older adults’ social media use is positively associated with their subjective well-being, with perceived social support playing a partial mediating role: forwarding behavior exerts its influence by promoting relationship maintenance. This proactive behavior of initiating social interaction through information transmission helps older adults receive timely replies, positive comments, and likes as positive feedback (Wang and Xu, 2024). According to social presence theory, the interactivity of digital media and confirmation of expectations for timely feedback are key elements for perceiving others’ presence in virtual environments (Tu, 2001). Such feedback makes older adults experience feelings of being cared for and heard, satisfying their needs for attention and emotional support, thereby strengthening social connection (Feeney and Collins, 2015), and corresponding to higher perceived social support levels and subjective well-being. Based on the above, this study proposes the following research hypothesis:

*H*3: Social support mediates the association between social media use and subjective well-being among older adults.

Future time perspective may also serve as an antecedent variable for how social media use affects social support acquisition. As mentioned earlier, social media use affects people’s perception and insight of time, and future time perspective also influences people’s social support. For example, Maki et al. (2016) believed that future time perspective is positively correlated with prosocial behavior and can positively predict individual voluntary behavior, helping behavior, and social support provision tendencies. Casu et al. (2020), in a study covering 1,256 adults, also found that both future time perspective dimensions of “remaining time” and “remaining opportunities” were significantly positively correlated with received social support and provided social support, meaning that individuals with more positive future assessments reported higher levels of social support perception. Recent research shows that future time perspective and social support are both associated with subjective well-being among older adults. These findings suggest that social media use may be linked to older adults’ subjective well-being by first expanding their future time perspective, which in turn may relate to more active engagement in social interaction and relationship maintenance, thereby corresponding to higher levels of received social support and, ultimately, greater subjective well-being. Although the above reasoning is logically plausible, no empirical research has directly tested this complete chain mediation pathway. In view of this, this study proposes the following research hypothesis:

*H*4: Future time perspective and social support play a chain mediating role in the association between social media use and subjective well-being among older adults.

The hypothesized directional relationships in this study are grounded in Socioemotional Selectivity Theory (SST), which provides clear theoretical bases for the proposed sequence. SST posits that future time perspective serves as a core motivational mechanism that regulates social goal selection and, consequently, social behavioral patterns (Carstensen, 2006; Lang and Carstensen, 2002). This theoretical logic establishes a clear conceptual sequence: temporal cognition (future time perspective) precedes and shapes social resource acquisition (social support), rather than the reverse. Furthermore, social media use is conceptualized as a behavioral antecedent—a daily digital participation behavior—that may influence both temporal cognition and social resources, providing a theoretically coherent rationale for placing it as the exogenous variable in the model.

## 3 Methods

### 3.1 Survey method and research participants

Based on the statistical data from the Seventh National Population Census, this study first determined the population distribution proportions of 23 provinces, 5 autonomous regions, and 4 municipalities nationwide, and calculated target sample sizes for each provincial administrative unit accordingly. During the sampling process, samples were drawn sequentially at different administrative levels including cities, districts/counties, townships/towns/streets, and communities/villages, followed by further sample screening at the individual level based on quota gender and age attributes. To ensure reasonable sample distribution across units at all levels, this study employed stratified randomization methods, allocating samples to corresponding-level units according to predetermined proportions. Under strict implementation of the above sampling criteria, a total of 5,200 questionnaires were distributed between November 1, 2025 and December 1, 2025. After collection, questionnaire validity was analyzed, yielding 4,872 valid questionnaires with an effective rate of 93.7%.

According to Article 2 of the Law of the People’s Republic of China on Protection of the Rights and Interests of the Elderly, “older adults” refers to citizens aged 60 and above. Therefore, the analytical sample was limited to individuals aged ≥60 at recruitment. Inclusion criteria: ①Age ≥60; ②Chinese nationality; ③Chinese permanent residents (annual time away ≤1 month); ④Voluntary participation in research with informed consent; ⑤Able to complete online questionnaire surveys independently or with investigator assistance; ⑥Understanding of the meaning expressed by each questionnaire item.

Exclusion criteria: ①Unconscious or mentally abnormal individuals; ②Currently participating in other similar research projects; ③Unwilling to cooperate.

At least one investigator or investigation team was recruited for each city, with each investigator responsible for collecting 30–90 questionnaires and each investigation team responsible for collecting 100–200 questionnaires. Investigators used the online questionnaire platform^1^ to distribute questionnaires face-to-face to the public in their respective areas. Respondents answered by clicking links, with informed consent obtained during surveys and questionnaire numbers input by investigators. If respondents had thinking ability but insufficient action capability to answer questionnaires, investigators conducted one-on-one inquiries and answered on their behalf. After questionnaire collection, two people conducted back-to-back logic checks and data screening. Sample characteristics are shown in Table 1.

**Table 1:** Demographic variable analysis (*N* = 4,872).

Variable	Category	Number (n)	Percentage (%)
Gender	Male	2,073	43%
	Female	2,799	57%
Age	60–65 years	2,827	58%
	66–70 years	914	19%
	71–80 years	755	16%
	81 + years	376	7%
Residence	Urban	3,393	70%
	Rural	1,479	30%
Education	Primary school and below	1,152	24%
	Junior high school	1,108	23%
	Senior high school	1,119	24%
	Junior college	667	14%
	Undergraduate	604	13%
	Master’s degree and above	222	3%
Marital status	Married	4,128	85%
	Divorced	53	1%
	Widowed	544	11%
	Single	147	3%
Monthly income	No income	790	16%
	1,000 yuan and below	437	9%
	1,001–3,000 yuan	1,473	30%
	3,001–5,000 yuan	1,302	27%
	5,001–10,000 yuan	622	13%
	Above 10,000 yuan	248	5%
Occupation	Public servants	864	18%
	Enterprise managers	917	19%
	Workers	971	20%
	Farmers	1,228	25%
	Individual entrepreneurs	341	7%
	Others	551	11%

Among 4,872 research participants, gender distribution was relatively balanced with slightly more females than males: 2,073 males (43%) and 2,799 females (57%). From age structure, participants were mainly young-old adults aged 60–65, totaling 2,827 (58%); 914 aged 66–70 (19%); 755 aged 71–80 (16%); and 376 aged 81 and above (7%). This age distribution well reflected the compositional characteristics of different age stages within older adult groups. In residential distribution, urban older adults totaled 3,393 (70%) and rural older adults 1,479 (30%), with urban sample proportions higher than rural, showing good geographical representation. Regarding education levels, samples covered all education levels from primary school and below to master’s degree and above: primary school and below 1,152 (24%), junior high school 1,108 (23%), senior high school 1,119 (24%), junior college 667 (14%), undergraduate 604 (13%), and master’s degree and above 222 (3%). Overall, participants’ education levels were relatively dispersed, mainly at secondary education levels. For marital status, married individuals comprised the vast majority at 4,128 (85%), divorced 53 (1%), widowed 544 (11%), and single 147 (3%). This distribution aligned with general marital characteristics of older adult groups, with the trend of increasing widowhood proportions with age also reflected. From monthly income levels, 790 had no income (16%), 437 earned 1,000 yuan and below (9%), 1,473 earned 1,001–3,000 yuan (30%), 1,302 earned 3,001–5,000 yuan (27%), 622 earned 5,001–10,000 yuan (13%), and 248 earned above 10,000 yuan (5%). Sample income distribution showed characteristics of more in the middle and fewer at both ends, mainly middle-income groups. For occupational distribution, public servants 864 (18%), enterprise managers 917 (19%), workers 971 (20%), farmers 1,228 (25%), individual entrepreneurs 341 (7%), and other occupations 551 (11%). Overall, this study’s sample had reasonable distribution across demographic variables including gender, age, residence, education level, marital status, monthly income, and occupation, providing a reliable data foundation for subsequent analysis.

### 3.2 Questionnaire design and variable measurement

The questionnaire design process was as follows: First, combining research-determined content, extensive relevant literature was collected, summarized, and organized to understand previous researchers’ questionnaire design situations and determine research questions and variables. Second, questions were reasonably set and categorized to obtain the initial questionnaire. Third, a pre-survey test was conducted, and based on pre-survey results, questionnaire validity and question reasonableness were tested, with further adjustments made accordingly to obtain the final questionnaire for formal survey use. The final distributed questionnaire mainly involved two parts: Part One demographic variables: mainly understanding basic information about survey participants. Part Two: measurement of various variables.

#### 3.2.1 Social media use scale

This study adopted the Social Media Use Integration Scale (SMUIS) developed by Jenkins-Guarnieri et al. (2013) to assess participants’ digital media use behavior. The scale includes two dimensions: social integration and emotional connection (6 items) and integration into social routines (4 items), using 5-point Likert scoring (1 = strongly disagree, 5 = strongly agree). Higher total scores indicate stronger dependence and emotional investment in social media. It should be noted that the SMUIS captures the overall degree of social media integration into daily life and emotional engagement, rather than distinguishing between specific usage patterns (e.g., active use such as posting and commenting versus passive use such as browsing). While this holistic approach is appropriate for examining the general association between social media engagement and well-being, it does not permit differentiation of effects attributable to distinct usage behaviors. This measurement limitation is further addressed in the Discussion section. Confirmatory factor analysis showed internal consistency coefficient *α* = 0.875 for this study’s sample, with Cronbach’s α values of 0.827 and 0.866 for subdimensions “social integration and emotional connection” and “integration into social routines” respectively, indicating excellent scale reliability.

#### 3.2.2 Subjective well-being scale

This study used the WHO Well-Being Index to measure participants’ subjective well-being levels (Topp et al., 2015). The WHO-5 Well-Being Index, published by the World Health Organization in 1998, is one of the most widely used questionnaires for assessing subjective psychological well-being. This measurement uses Likert 5-point scoring, with options ranging from never (0 points) to all the time (5 points). Initial scoring is the sum of 5 item response values, with total score ranges from 0 to 25 points. Higher scores indicate higher well-being index, meaning higher subjective well-being. The Cronbach’s *α* coefficient for this scale in this study was 0.813.

#### 3.2.3 Social support satisfaction scale

The Questionnaire on the Frequency of and Satisfaction with Social Support (QFSSS) was used to measure older adults’ satisfaction with received and provided social support, specifically covering different support types (emotional support, instrumental support, and informational support) given and received from different targets in their social networks (partners, family members, friends, and community) (García-Martín et al., 2016). The questionnaire includes 12 items about received support and 12 items about provided support, using 5-point scoring from 1 point (very dissatisfied) to 5 points (very satisfied). Participants needed to assess their satisfaction with received and provided social support (based on support sources and types). This study used average scores for social support receipt and provision calculated based on three support types and four support sources. The total scale’s Cronbach’s *α* coefficient was 0.937. Example items for social support receipt satisfaction include: “You feel loved, listened to, and someone is willing to listen when you need to confide and express emotions” (emotional support from partners); “Your partner does things for you, such as helping with daily housework or childcare” (instrumental support from partners). Example items for social support provision satisfaction include: “You provide useful advice or information to solve their questions, problems, or daily affairs” (informational support to friends); “You are happy to do specific things for them, such as helping with daily housework or childcare” (informational support to community members). The Cronbach’s α coefficient for this scale in this study was 0.825.

#### 3.2.4 Future time perspective scale

Future time perspective refers to individuals’ perception and cognition of their remaining lifetime. This study adopted the Future Time Perspective Scale developed by Lang and Carstensen (2002) for measurement. The scale contains 10 items aimed at examining whether individuals perceive their remaining lifetime as limited or unlimited, using 5-point Likert scale scoring from 1 point (strongly disagree) to 5 points (strongly agree). Older participants were required to assess their agreement with 10 statements. Typical items include: “Many opportunities await me in the future,” “I have ample time to make new plans,” “My future seems unlimited”. This study used average scores of 10 items for analysis, with 3 reverse-worded items (such as “I feel that time remaining is limited”, “As I grow older, I begin to feel that time is limited”) reverse-coded. Higher scores indicate that individuals perceive more abundant future remaining time. The Cronbach’s α coefficient for this scale in this study was 0.891.

## 4 Results

### 4.1 Common method variance testing

This study employed Harman’s single-factor test to examine possible common method variance. Based on data analysis of 4,872 valid samples, without factor rotation, six common factors with eigenvalues greater than 1 were extracted, with the primary factor’s variance explanation rate of 31.772%. This value was below the conventional 40% threshold, providing preliminary evidence that common method variance does not represent a dominant source of variance in the data. However, it should be noted that Harman’ s single-factor test has recognized limitations in sensitivity and may not fully capture the extent of common method bias (Podsakoff et al., 2003). As all variables in this study were assessed through self-report measures collected at a single time point, the possibility of some inflation of observed associations due to shared method variance cannot be entirely excluded. To further mitigate this concern, confirmatory factor analysis was conducted to verify the discriminant validity of the four-factor measurement model (see Section 4.2), and the results supported adequate construct separation. Nevertheless, future research should consider procedural remedies and statistical remedies to more rigorously address this methodological limitation.

### 4.2 Confirmatory factor analysis

This study used AMOS 26.0 statistical software to conduct confirmatory factor analysis on four core latent variables based on 4,872 sample data. Model fit test results are detailed in Table 2. Analysis results showed: the four-factor model chi-square to degrees of freedom ratio (χ^2^/df) = 2.613 (less than critical value 3), root mean square error of approximation (RMSEA) = 0.036 (below 0.08 threshold), and comparative fit index (CFI), Tucker-Lewis index (TLI), and goodness of fit index (GFI) all reached above 0.90. Various fit indices not only fully met statistical requirements, but model comparison found this model showed superior fit effects compared to other competitive alternative models, indicating high discriminant validity in this study. Multicollinearity among predictors was evaluated using variance inflation factors (VIF), calculated as VIF = 1/(1 − R^2^), where R^2^ represents the coefficient of determination obtained by regressing each predictor on the remaining predictors (Hair et al., 2019). All VIF values ranged from 1.05 to 1.07, well below the commonly adopted threshold of 10, indicating no multicollinearity concerns among the variables in the present model.

**Table 2:** Confirmatory factor analysis.

Model	χ^2^/df	RMSEA	CFI	TLI	GFI
Four-factor model	2.613	0.036	0.958	0.948	0.972
Three-factor model	3.574	0.072	0.915	0.897	0.905
Two-factor model	4.826	0.198	0.783	0.761	0.774
Single-factor model	5.937	0.312	0.687	0.661	0.672

### 4.3 Descriptive statistical analysis and correlation analysis

Mean values, standard deviations, and correlation coefficient analysis results for each variable are shown in Table 3. Analysis results in dictate that social media use is significantly positively correlated with social support (*r* = 0.234, *p* < 0.01), significantly positively associated with future time perspective (*r* = 0.193, *p* < 0.01), and significantly positively correlated with subjective well-being (*r* = 0.164, *p* < 0.01). At the social support level, it is significantly positively correlated with future time perspective (*r* = 0.221, *p* < 0.01) and significantly positively associated with subjective well-being (*r* = 0.237, *p* < 0.01). Additionally, there is moderate-strength significant positive correlation between future time perspective and subjective well-being (*r* = 0.357, *p* < 0.001). The above correlation patterns preliminarily reveal positive association pathways between variables, providing empirical basis for subsequent structural equation modeling and mediation effect testing.

**Table 3:** Descriptive statistics and correlation analysis.

Variable	M	SD	Social media use	Social support	Future time perspective	Subjective well-being
Social media use	2.817	0.857	1			
Social support	2.779	0.432	0.234**	1		
Future time perspective	3.273	0.354	0.193**	0.221**	1	
Subjective well-being	2.315	0.583	0.264**	0.237**	0.357***	1

*, *, *** represent *p* < 0.05, *p* < 0.01, *p* < 0.001, respectively.

### 4.4 Main effects and mediation effects testing

**Figure 1:**
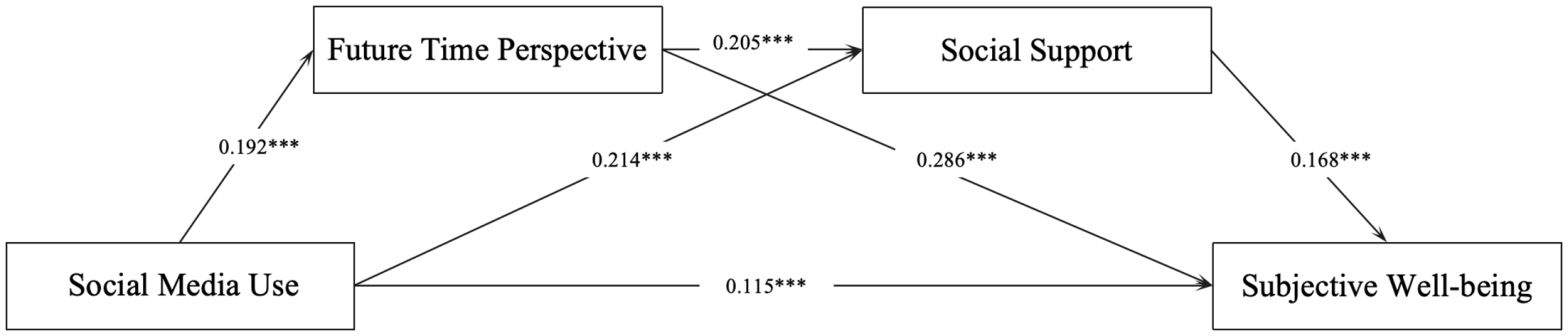
Structural equation model path coefficients.

Based on the conceptual model, this study constructed a structural equation model with social media use, social support, future time perspective, and subjective well-being as core variables. In testing the hypothesized relationships, social media use had a significant positive direct effect on future time perspective (*β* = 0.192, *p* < 0.001), on social support (*β* = 0.214, *p* < 0.001), and on subjective well-being (*β* = 0.115, *p* < 0.001). Further analysis revealed that future time perspective was significantly and positively associated with social support (*β* = 0.205, *p* < 0.001) and with subjective well-being (*β* = 0.286, *p* < 0.001). Additionally, social support was significantly and positively associated with subjective well-being (*β* = 0.168, *p* < 0.001). In summary, all hypothesized pathways in the model reached significance levels, with the direction of each association consistent with theoretical expectations. Path coefficients and significance levels for each variable relationship are detailed in Table 4 and illustrated in Figure 1.

**Table 4:** Model path relationship hypothesis testing results.

Path relationship	*β*	S. E.	C. R.	*p*-value	Conclusion
Social Media Use → Future Time Perspective	0.192	0.019	9.579	< 0.001	Supported
Social Media Use → Social Support	0.214	0.017	12.176	< 0.001	Supported
Social Media Use → Subjective Well-being	0.115	0.015	8.267	< 0.001	Supported
Future Time Perspective → Social Support	0.205	0.021	9.333	< 0.001	Supported
Future Time Perspective → Subjective Well-being	0.286	0.020	14.1	< 0.001	Supported
Social Support → Subjective Well-being	0.168	0.019	8.158	< 0.001	Supported

Based on the research conceptual model, this paper hypothesizes that future time perspective and social support have mediation effects between social media use and subjective well-being among older adults, analyzed and verified through AMOS 28.0. Using bias-corrected non-parametric percentile Bootstrap method (5,000 resamples), mediation effects of future time perspective and social support were verified. Table 5 shows standardized effect estimates and 95% confidence intervals for each indirect pathway. If confidence intervals do not contain 0, mediation effects are significant. According to Table 5 data, 95% confidence intervals for all three indirect pathways do not contain 0, indicating that independent mediation effects of future time perspective, independent mediation effects of social support, and their chain mediation effects all reach significant levels. Specifically, the mediation effect value of future time perspective is 0.055, with 95% confidence interval [0.043, 0.067]; the mediation effect value of social support is 0.032, with 95% confidence interval [0.027, 0.046]; the chain mediation effect value of future time perspective and social support is 0.010, with 95% confidence interval [0.006, 0.014]. Therefore, Hypotheses 2, 3, and 4 of this study are all empirically supported.

**Table 5:** Mediation path relationship testing results.

Path	Standardized path coefficient	Standard error	95% confidence interval
Social Media Use → Future Time Perspective → Subjective Well-being	0.055	0.006	[0.043, 0.067]
Social Media Use → Social Support → Subjective Well-being	0.036	0.005	[0.027, 0.046]
Social Media Use → Future Time Perspective → Social Support → Subjective Well-being	0.010	0.002	[0.006, 0.014]

## 5 Discussion

### 5.1 Direct effects of social media use on subjective well-being among older adults

The results of the structural equation modeling indicate that social media use has a significant positive direct effect on subjective well-being among older adults, consistent with prior research documenting favorable links between social media engagement and older adults’ physical and psychological health (Cornejo et al., 2013). This finding lends support to the digital transformation logic of active aging, suggesting that digital participation serves not only as a channel for information acquisition but also as a domain for psychological resource supplementation and life meaning reconstruction.

Several mechanisms may account for this positive association. First, social media transcends spatial–temporal constraints, restructuring social connection patterns for older adults. The bidirectional interaction and instant feedback afforded by platforms such as WeChat enable older adults to sustain existing relationships and broaden social circles (Simons et al., 2023), thereby alleviating loneliness (Khalaila and Vitman-Schorr, 2018) and fostering a sense of belonging closely linked to well-being. Second, drawing on the Needs-Affordances-Features (NAF) model (Karahanna et al., 2018; Lian et al., 2023), social media’s functional affordances correspond to older adults’ basic psychological needs. Connectivity affordances address belongingness needs by digitally offsetting spatial isolation; functional affordances support competence through self-efficacy gained from mastering digital operations; and expressive affordances satisfy autonomy needs by enabling self-expression and restoring social visibility. The interplay of these need-fulfillment pathways collectively corresponds to subjective well-being (Wang and Han, 2025). Third, internet use expands older adults’ access to health information, with indirect implications for well-being. Enhanced health knowledge improves self-care capabilities (Shoaib, 2023; Yuan, 2021), and media use is associated with well-being by helping older adults overcome limitations such as physical disabilities and social isolation (Dal Cin et al., 2023; Tirado-Morueta et al., 2023).

However, the positive association documented here should not be interpreted as evidence that social media use is unconditionally beneficial for older adults. The differential susceptibility to media effects model (Valkenburg and Peter, 2013) suggests that identical media exposure may produce divergent outcomes depending on individual predispositions and situational factors. For older adults specifically, passive consumption may trigger unfavorable social comparisons (Verduyn et al., 2017), exposure to health-related misinformation may heighten anxiety, and lower digital literacy may lead to frustration rather than psychological benefits. As the SMUIS employed in this study captures general social media integration rather than specific usage modes, the observed net positive association likely reflects the average across heterogeneous usage experiences. Future research should examine under what conditions and for whom social media use is associated with positive versus negative well-being outcomes.

Moreover, these direct associations alone cannot fully account for the relationship, as prior research suggests the link is often indirect, operating through specific psychological and social mechanisms (Nam, 2021; Gu et al., 2025). The following sections examine the mediating roles of future time perspective and social support.

### 5.2 Mediation effects of future time perspective

This study found that future time perspective plays a significant mediating role in the association between social media use and subjective well-being among older adults. This finding reveals a psychological mechanism that has received limited attention in prior research: social media use may be linked to subjective well-being through its association with older adults’ perception and evaluation of remaining lifetime.

Socioemotional Selectivity Theory (SST) offers a plausible explanation for this relationship. The theory emphasizes that future time perspective is inherently malleable, with age serving as a proximal predictor rather than a determinant (Carstensen, 2021). Empirical evidence confirms that situational factors can produce fluctuations in time perception (Fredrickson and Carstensen, 1990; Katana et al., 2020). Accordingly, social media—as a digital technology deeply embedded in daily life—may function as a situational factor that broadens older adults’ information accessibility and social participation, thereby expanding their perception of future opportunities. Specifically, social media may “stretch” older adults’ psychological time horizons through the continuous provision of novel social experiences and cognitive stimulation, which in turn is positively associated with subjective well-being.

Brothers et al. (2019) distinguished future time perspective into threedimensions: perceived extension, perceived opportunity, and perceivedconstraints. Social media provides older adults with information acquisition and social interaction channels that transcend physical limitations, enabling continuous exposure to new knowledge, activities, and possibilities. This sustained cognitive stimulation and social engagement may help attenuate negative perceptions of “time running out” while strengthening positive assessments of future opportunities. In other words, social media use may be indirectly associated with subjective well-being through its links to enhanced time extension and opportunity perception and reduced constraint perception. This interpretation resonates with Barber and Tan’s (2018) finding that older adults holding negative aging self-concepts tend to perceive more constrained future possibilities, with negative emotional states mediating this relationship. By extension, social media use may play a countervailing role: when older adults successfully accomplish communicative, informational, and relational tasks on digital platforms, these positive experiences may foster more favorable aging self-concepts, thereby broadening positive expectations for the future.

SST also implies a deeper psychological process: the enhancement of time value (Chu and Carstensen, 2022). The theory’s core assumption is that when individuals perceive contraction of the time horizon, they not only adjust goal priorities but also develop a deeper appreciation for remaining time (Growney et al., 2025). Chu and Carstensen (2023) conceptualize this state as “time savoring”—the ability to focus on, appreciate, and amplify positive experiences. In the temporal dimension, time savoring manifests as immersion in the present accompanied by heightened awareness and appreciation of time’s passage, encompassing both recognition of time’s finitude and deep emotional investment in remaining time. From this theoretical vantage point, social media use may activate this time savoring process. For instance, when older adults conduct video calls with relatives and friends through WeChat, they may experience a dual temporal sensation: instant digital connections foster the expectation that future emotional bonds remain viable, extending the psychological time horizon, while the real-time interactions and emotional exchanges cultivate deep appreciation for present companionship. Such experiences may guide older adults from a quantitative orientation of “how much time remains” toward a qualitative awareness of “how to spend remaining time,” thereby uncovering richer meaning within finite life horizons.

### 5.3 Mediation effects of social support

Whereas future time perspective operates at the cognitive level, social support functions as a social resource-level mediator in the association between social media use and subjective well-being among older adults. The results indicate a significant mediating role of social support, consistent with findings by Nam (2021) and Gu et al. (2025), who identified social support as a key linking mechanism between internet use and well-being.

From a theoretical standpoint, the mediating role of social support can be interpreted through the classic dual-process model proposed by Cohen and Wills (1985). The main effect model posits that social support directly contributes to physical and mental health by providing individuals with a sense of belonging, self-worth confirmation, and positive social role identity (Cobb, 1976; Broadhead et al., 1983). For older adults, post-retirement contraction of social networks often leads to role loss and belongingness crises (Song et al., 2021; Nie et al., 2002). Social media use may offer convenient channels for rebuilding and maintaining social connections—such as communicating with relatives and friends via WeChat, participating in group interactions, and sharing life information—thereby enhancing perceived social support levels with corresponding benefits for subjective well-being. The stress-buffering model, in contrast, argues that social support primarily functions protectively when individuals face stressful events, preserving psychological health by regulating stress appraisals or attenuating stress responses (Cohen and Wills, 1985). Older adults frequently encounter adverse life events including health decline, bereavement, and economic hardship, while emotional comfort, informational guidance, and practical assistance obtained through social media may effectively buffer these stressors’ negative associations with well-being. The present findings provide indirect empirical support for both mechanisms.

At the practical level, these findings suggest that encouraging active rather than passive social media use, optimizing age-friendly platform design for interactive functions, and promoting timely family responsiveness to older adults’ online interactions may collectively enhance perceived social support and, in turn, well-being.

### 5.4 Chain mediation effects of future time perspective and social support

The preceding sections examined future time perspective and social support as independent mediators. A more noteworthy finding of this study is that these two variables do not function merely as parallel mediators but appear to constitute a progressive chain mediation pathway. The key to interpreting this chain pattern lies in addressing a question that the previous sections left unanswered: why might a sequential relationship exist between future time perspective and social support?

Lang and Carstensen (2002) found that individuals who perceive an open future time horizon place high value on social acceptance goals, including having friends who accept them, maintaining intimate and trusting relationships, and receiving advice on important decisions. Notably, these social acceptance goals overlap substantially with the core elements of social support as defined by Cobb (1976)—being cared for, loved, respected, and belonging to reciprocal networks. Pursuing social acceptance goals functionally approximates creating relational conditions conducive to obtaining social support. When older adults attach greater value to social acceptance due to a more expansive future time perspective, they may be more inclined to actively maintain relationships, seek assistance, and respond to others’ needs through social media. Casu et al. (2020) findings corroborate this reasoning: individuals with a more open future time perspective not only received more social support but also provided more support to others, forming reciprocal exchange patterns.

Additionally, the present findings engage in theoretical dialog with Zhang et al. (2019) research, which demonstrated that social network size is associated with well-being through future time perspective—that is, social structural resources relate to well-being via temporal cognition. The present study reveals a complementary pathway: future time perspective is associated with well-being through social support—that is, temporal cognition corresponds to social resource acquisition. Taken together, both studies suggest a reciprocal relationship between future time perspective and social relationships. The present study’s contribution lies in introducing social media use as a novel digital-age antecedent at the front end of this reciprocal process, while extending SST’s traditional emphasis on social network structural characteristics to the functional dimension of social support at the back end.

The chain mediation pattern suggested by this study can be interpreted as follows: social media use, as a daily digital participation behavior, is associated with more positive subjective aging perceptions and expanded assessments of future opportunities among older adults, which in turn correspond to greater investment in social interaction and relationship maintenance, thereby relating to higher levels of perceived social support and, ultimately, to enhanced subjective well-being through the direct benefits and stress-buffering functions of social support.

Importantly, the findings of this study should be interpreted within the Chinese cultural context. Several culturally specific factors may shape the observed associations. First, Chinese culture emphasizes filial piety and intergenerational solidarity, which may amplify the role of family-based social support in well-being relative to Western individualistic societies, where peer and community support may play comparatively larger roles (Mohd et al., 2019; Peng et al., 2015). Second, the dominant social media platform among Chinese older adults is WeChat, which uniquely integrates messaging, social networking, and mobile payment functions within a family-centered communication architecture. This platform ecology differs substantially from the fragmented platform landscape prevalent in Western countries (e.g., separate use of Facebook, WhatsApp, and Instagram), potentially influencing how social media use relates to social support. Third, Chinese cultural values regarding aging—including respect for elders and collective family responsibility—may shape how future time perspective operates differently than in cultures with more individualistic orientations toward aging.

The theoretical contributions of this study are threefold. First, it broadens the research perspective on antecedent variables of future time perspective. SST has traditionally focused on the consequences of future time perspective while devoting limited attention to identifying daily experiences that may expand it. The present study documents a positive association between social media use and future time perspective among older adults, providing preliminary evidence for identifying positive antecedents of future time perspective and extending SST’ s theoretical claims regarding its plasticity from laboratory-based negative manipulations to everyday digital life contexts. Second, this study extends SST’s social implications from network structure to network function. SST has traditionally examined how future time perspective relates to social network morphology; the present findings indicate that future time perspective is also significantly associated with the functional social support older adults actually receive, suggesting that the influence pathways linked to future time perspective encompass not only relational structures but also support resource acquisition. Third, this study offers a time-motivational explanation for the relationship between digital technology and older adults’ well-being, supplementing an important cognitive dimension to understanding how digital engagement relates to psychological outcomes in later life.

## 6 Limitations

Several limitations of this study must be acknowledged. First, the cross-sectional design precludes causal inference; all observed relationships should be interpreted as associations rather than causal effects. Although the proposed directional model is grounded in Socioemotional Selectivity Theory, alternative causal directions (e.g., higher well-being leading to greater social media use) or reciprocal relationships cannot be ruled out. Future research should employ longitudinal panel designs or quasi-experimental methods to establish temporal precedence and strengthen causal claims. Second, all variables were measured through self-report instruments, which may introduce common method bias. Future studies should incorporate objective measures of social media use (e.g., digital trace data, screen time logs) and multi-informant assessments of social support to mitigate this concern. Third, the sample is restricted to older adults in China, where cultural factors such as filial piety, intergenerational solidarity, and the dominance of WeChat as an integrated super-app may shape the observed associations in ways that differ from Western or other cultural contexts. The particular significance of family support in Chinese culture and the unique platform ecology of WeChat limit the generalizability of these findings. Cross-cultural replication studies are needed to determine whether the chain mediation mechanism operates similarly across different cultural and technological environments. Fourth, social media use measurement focused on integration degree and emotional connection (using the SMUIS) without distinguishing between different usage patterns such as active use (e.g., posting, commenting, messaging) versus passive use (e.g., browsing, lurking). Research has shown that active and passive social media use may have differential associations with well-being (Verduyn et al., 2017). Future research should employ more fine-grained behavioral measures to examine whether specific types of social media activities drive the observed associations. Fifth, online recruitment may have excluded older adults who do not use the internet, creating potential selection bias that limits generalizability to the broader older adult population. Sixth, the present study did not statistically control for potential confounding variables such as physical health status, digital literacy, and living arrangements in the structural equation model. Although the sample demonstrated reasonable demographic variation (see Table 1), the observed associations may partly reflect the influence of unmeasured third variables. Future studies should incorporate relevant covariates and consider approaches such as propensity score matching to provide more precise estimates of the hypothesized pathways.

## 7 Conclusion

Based on socioemotional selectivity theory and social support theory, this study constructed and tested a theoretical model examining the associations among social media use, future time perspective, social support, and subjective well-being among older adults. Through data analysis, the following main conclusions were drawn: First, social media use is significantly and positively associated with subjective well-being among older adults, suggesting that digital participation may serve as an important psychological resource for active aging. Second, future time perspective plays an independent mediating role: social media use is linked to older adults’ more positive perceptions of future opportunities through cognitive stimulation and social experiences, and this perception is in turn associated with higher well-being. Third, social support also serves as an independent mediator, supporting social media’s functional value as a channel for social support acquisition. Fourth, future time perspective and social support constitute a chain mediation pathway: social media use is associated with expanded future time cognition, which corresponds to greater investment in social interaction and is associated with higher levels of perceived social support and, ultimately, with enhanced subjective well-being. This progressive linkage mechanism reveals the internal logic of temporal cognition corresponding to social resource acquisition, which is this study’s core theoretical contribution.

This study provides multi-level intervention targets for promoting digital well-being among older adults. At the policy level, governments should integrate digital literacy training into community-based aging services, with curricula specifically designed to enhance older adults’ competence in using social media for active social interaction rather than passive consumption. At the platform level, technology companies should prioritize age-friendly design that optimizes interactive communication functions—such as simplified video calling interfaces and intuitive group messaging—which may help lower technical barriers to social participation. At the family level, members should actively respond to interaction behaviors initiated by older adults on social media, strengthening their sense of social presence and perceived social support through timely feedback. At the community level, local organizations could establish digital mentoring programs pairing younger volunteers with older adults to facilitate meaningful social media engagement. These multi-level strategies collectively aim to enhance older adults’ social support perception and future time perspective through digital participation, which may in turn contribute to subjective well-being.

## Data Availability

The raw data supporting the conclusions of this article will be made available by the authors, without undue reservation.

## Footnotes

1
https://www.wjx.cn/
